# The Underlying Pathogenesis of Neurovascular Compression Syndromes: A Systematic Review

**DOI:** 10.3389/fnmol.2022.923089

**Published:** 2022-07-04

**Authors:** Bartosz Szmyd, Julia Sołek, Maciej Błaszczyk, Jakub Jankowski, Paweł P. Liberski, Dariusz J. Jaskólski, Grzegorz Wysiadecki, Filip F. Karuga, Agata Gabryelska, Marcin Sochal, R. Shane Tubbs, Maciej Radek

**Affiliations:** ^1^Department of Neurosurgery, Spine and Peripheral Nerve Surgery, Medical University of Lodz, Lodz, Poland; ^2^Department of Pathology, Chair of Oncology, Medical University of Lodz, Lodz, Poland; ^3^Department of Molecular Pathology and Neuropathology, Medical University of Lodz, Lodz, Poland; ^4^Department of Neurosurgery and Neurooncology, Medical University of Lodz, Lodz, Poland; ^5^Department of Normal and Clinical Anatomy, Medical University of Lodz, Lodz, Poland; ^6^Department of Sleep Medicine and Metabolic Disorders, Medical University of Lodz, Lodz, Poland; ^7^Department of Neurosurgery and Ochsner Neuroscience Institute, Ochsner Health System, New Orleans, LA, United States; ^8^Department of Neurosurgery, Tulane University School of Medicine, New Orleans, LA, United States; ^9^Department of Neurology, Tulane University School of Medicine, New Orleans, LA, United States; ^10^Department of Structural and Cellular Biology, Tulane University School of Medicine, New Orleans, LA, United States; ^11^Department of Surgery, Tulane University School of Medicine, New Orleans, LA, United States; ^12^Department of Anatomical Sciences, St. George's University, St. George's, Grenada; ^13^University of Queensland, Brisbane, QLD, Australia

**Keywords:** neurovascular compression syndromes, neurovascular conflicts, trigeminal neuralgia, glossopharyngeal neuralgia, hemifacial spasm, pathogenesis

## Abstract

Neurovascular compression syndromes (NVC) are challenging disorders resulting from the compression of cranial nerves at the root entry/exit zone. Clinically, we can distinguish the following NVC conditions: trigeminal neuralgia, hemifacial spasm, and glossopharyngeal neuralgia. Also, rare cases of geniculate neuralgia and superior laryngeal neuralgia are reported. Other syndromes, e.g., disabling positional vertigo, arterial hypertension in the course of NVC at the CN IX-X REZ and torticollis, have insufficient clinical evidence for microvascular decompression. The exact pathomechanism leading to characteristic NVC-related symptoms remains unclear. Proposed etiologies have limited explanatory scope. Therefore, we have examined the underlying pathomechanisms stated in the medical literature. To achieve our goal, we systematically reviewed original English language papers available in Pubmed and Web of Science databases before 2 October 2021. We obtained 1694 papers after eliminating duplicates. Only 357 original papers potentially pertaining to the pathogenesis of NVC were enrolled in full-text assessment for eligibility. Of these, 63 were included in the final analysis. The systematic review suggests that the anatomical and/or hemodynamical changes described are insufficient to account for NVC-related symptoms by themselves. They must coexist with additional changes such as factors associated with the affected nerve (e.g., demyelination, REZ modeling, vasculature pathology), nucleus hyperexcitability, white and/or gray matter changes in the brain, or disturbances in ion channels. Moreover, the effects of inflammatory background, altered proteome, and biochemical parameters on symptomatic NVC cannot be ignored. Further studies are needed to gain better insight into NVC pathophysiology.

## Graphical Abstract

So far, proposed etiologies of neurovascular compression syndromes have limited explanatory scope. Therefore, we have examined the underlying pathomechanisms stated in the medical literature. The systematic review suggests that the anatomical and/or hemodynamical changes described are insufficient to account for NVC-related symptoms by themselves. They must coexist with additional factors such as affected nerves (e.g., demyelination, REZ modeling, vasculature pathology), nucleus hyperexcitability, white and/or gray matter changes in the brain, or disturbances in ion channels. Moreover, the effects of inflammatory background, altered proteome, and biochemical parameters on symptomatic neurovascular compression syndromes cannot be ignored in the future. 
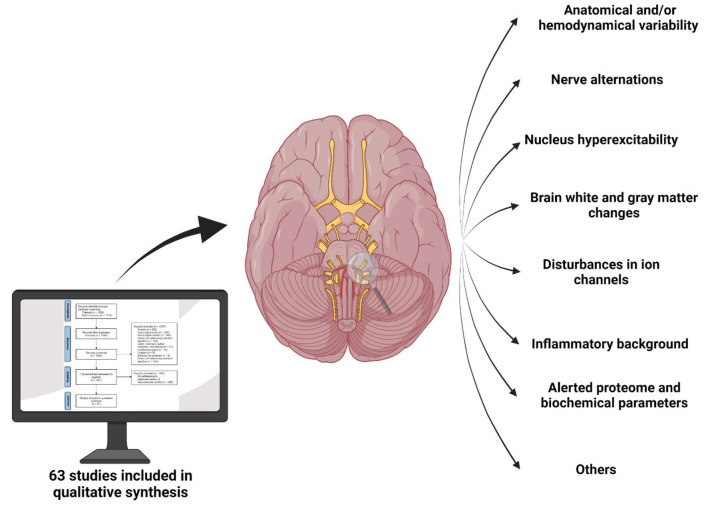

## Introduction

Neurovascular compression syndromes (NVC) are challenging disorders resulting from the compression of cranial nerves at the root entry/exit zone (REZ) also known as Redlich–Obersteiner's zone. Although the REZ is equated with the transition zone (TZ) between the central myelin produced by oligodendroglia and the peripheral myelin comprising Schwann cells, some publications have revealed differences between the two. Peker et al., who tested 100 trigeminal nerves from 50 cadavers, showed that on average the TZ is 3 mm from the REZ into the pons (Peker et al., 2006). This observation has a significant clinical consequence. During the surgery, it is important to visualize the compression of an appropriate vessel on the nerve in the TZ, e.g., in the situation in which TZ is retracted toward the pons, the neurosurgeon should look for conflict between vessel and pons rather than nerve.

Clinically, we can distinguish the following major NVC conditions: trigeminal neuralgia (TN), hemifacial spasm (HFS), and glossopharyngeal neuralgia. Also, rare cases of geniculate neuralgia and superior laryngeal neuralgia have been described (Greenberg, 2020). Disabling positional vertigo, arterial hypertension in the course of NVC at the CN IX-X REZ, and torticollis have insufficient clinical evidence for microvascular decompression (MVD; see Table 1). Nerve compression by a vessel is insufficient to cause NVC-related symptoms by itself. The pathomechanism leading to characteristic NVC-related symptoms remains unclear. Proposed etiologies have limited explanatory scope. Therefore, we have examined the underlying pathomechanisms described in the medical literature and subjected them to a systematic review.

**Table 1:** Typical offending vessel depending on specific neurovascular compression syndromes (NVC) in accordance with Greenberg (2020).

**Cranial nerve**		**Syndrome**	**Offending vessel**
**Number**	**Name**		
**Typical neurovascular compression syndromes**				
V	Trigeminal nerve	Trigeminal neuralgia	SCA (80-90%), remaining cases (10-20%): persistent primitive trigeminal artery, dolichoectatic basilar artery	
VII	Facial nerve	Hemifacial spasm	AICA (mainly), remaining cases: elongated PICA, SCA, a tortuous VA, the cochlear artery, a dolichoectatic basilar artery, AICA branches	
IX	Glossopharyngeal nerve	Glossopharyngeal neuralgia	PICA, VA	
**Rare cases**				
VII	Nervus intermedius	Geniculate neuralgia	AICA	
X	Vagus nerve	Superior laryngeal neuralgia	PICA, VA	
**Insufficient clinical evidence for MVD**				
VIII	Vestibulocochlear nerve	Disabling positional vertigo	AICA	NVC is a rare cause of vertigo. MVD should be limited to selected cases.
X	Vagus nerve	Arterial hypertension in the course of NVC at the CN IX-X REZ	PICA, VA	This topic has not been properly explored.
XI	Accessory nerve	Torticollis	VA, PICA (rarely)	Torticollis should be considered as focal dystonia.

*NVC were divided into three groups based on their significance: typical neurovascular compression syndromes, rare cases, and concepts of historical importance. AICA, anterior inferior cerebellar artery; MVD, microvascular decompression; PICA, posterior inferior cerebellar artery; SCA, superior cerebellar artery; VA, vertebral artery*.

## Materials and Methods

**Figure 1:**
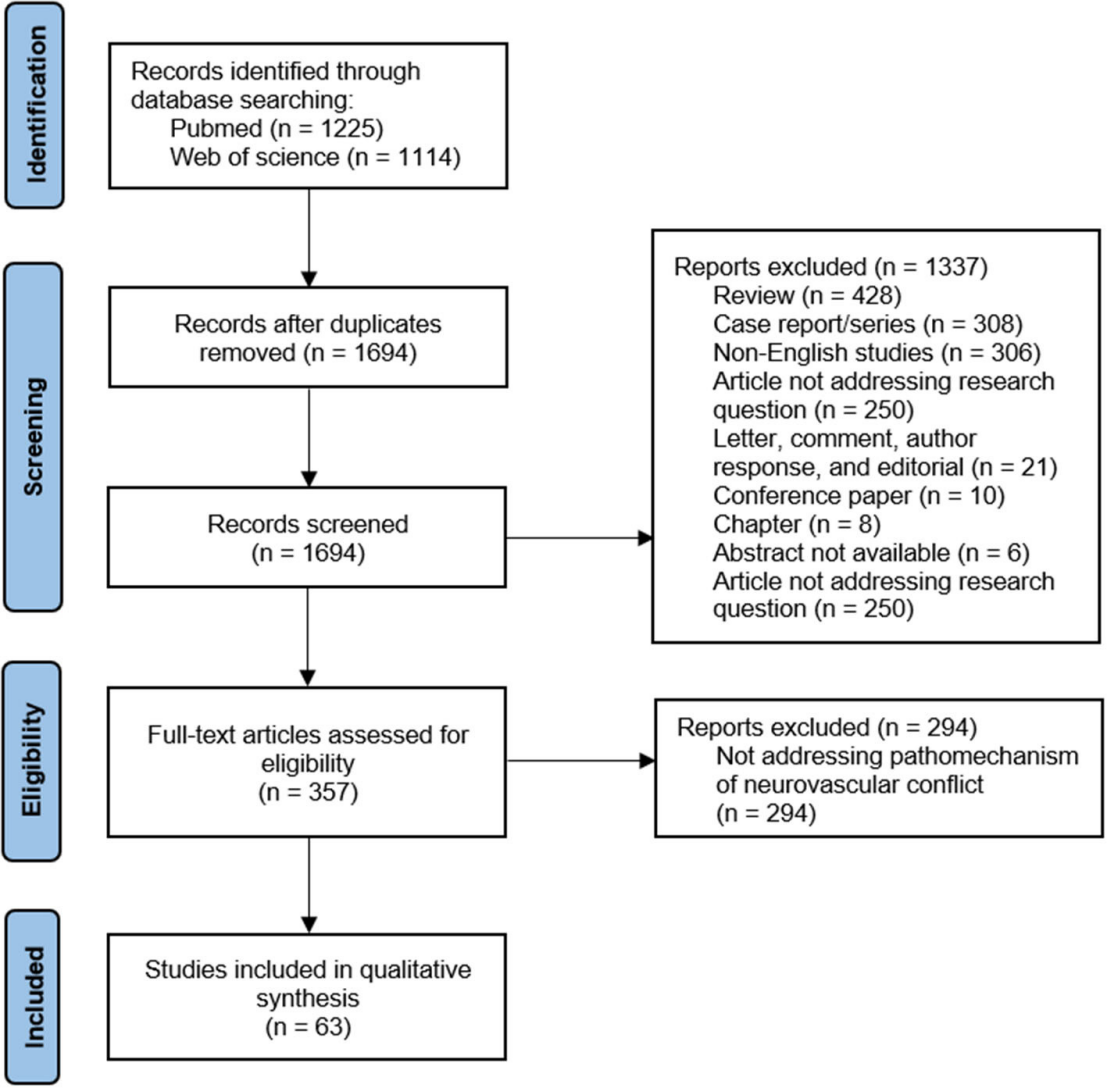
The flow-chart of publications included process.

Two of us (BSz and JS) screened all the relevant original English language papers published in the Pubmed and Web of Science databases before 2 October 2021. We decided to include all available subtypes of original papers to maximize the coverage of possible pathomechanisms of NVC. The exact queries are given in the Supplementary Materials. We obtained 1694 articles after eliminating duplicates. These records were double screened. In the case of any discrepancies between the two authors extracting data, the final decision was made by the senior author (MR). Only 357 original papers potentially pertaining to the pathogenesis of NVC were enrolled in the full-text assessment for eligibility (see Figure 1), and 63 of these were included in the final analysis.

## The Influence of Anatomical and Hemodynamical Changes on the Course of NVC

### Anatomical Variability

Selected anatomical variabilities such as a duplicate posterior inferior cerebellar artery, a short basilar artery, and an aberrant arterial course can affect the course of a NVC (Karki et al., 2019). Among other known factors, we can distinguish narrowness of the posterior fossa, a small petrous angle, and a sharp nerve-pontine angle.

Posterior fossa narrowness is an important example of the anatomical variabilities predisposing to NVC. This topic was explored by Kamiguchi et al., who measured the petrous angle and pons diameter index in 34 HFS patients. They observed a significantly smaller petrous angle combined with a significantly greater pons diameter index in the HFS patients than the control group. This anatomical variability led to more crowding of cranial nerves and vascular structures, which could contribute to symptomatic NVC (Kamiguchi et al., 1997).

Zhu et al. suggested that a small cross-sectional area of the cerebellopontine angle (CPA) could promote HFS pathogenesis. This hypothesis was corroborated by preoperative findings. Interestingly, these measurements can be considered predictors of the microvascular decompression (MVD) effect: they were significantly lower in the recurrent group than in the non-recurrent group (Zhu et al., 2020). This hypothesis is consistent with available literature (Dou et al., 2016; Cheng et al., 2017). Moreover, a crowded CPA space and a tendency toward attrition of the neurovascular components are considered likely to promote the occurrence of bilateral HFS (Dou et al., 2016).

Not only a small CPA but also a sharp nerve-pontine angle can lead to the NVC and also to the exacerbation of nerve degeneration (Pang et al., 2019; Zhu et al., 2020). From the clinical point of view, these anatomical factors are insufficient to cause NVC-related symptoms by themselves. As there are observed among many patients without NVC-related symptoms.

### Hemodynamical Factors

Lorenzoni et al. observed that the following factors increased the risk of TN: compression of the superior cerebellar, anterior inferior cerebellar, basilar or vertebral arteries, and of venous structures. Moreover, they observed nerve dislocation and/or distortion by the vessel in 32% of cases (Lorenzoni et al., 2012).

Anatomical variations and hemodynamical changes in the vertebrobasilar arterial system are important promoters of vascular compression among HFS patients (Kim et al., 2012; Wang et al., 2019a). The risk of HFS is greater among individuals with a dominant vertebral artery and characteristic direction of the vertebrobasilar junction (Park et al., 2015). Further, lateral deviation of the vertebral artery is significantly more frequent among HFS patients, with a relative risk of 8.44 (Guan et al., 2011).

**Figure 2:**
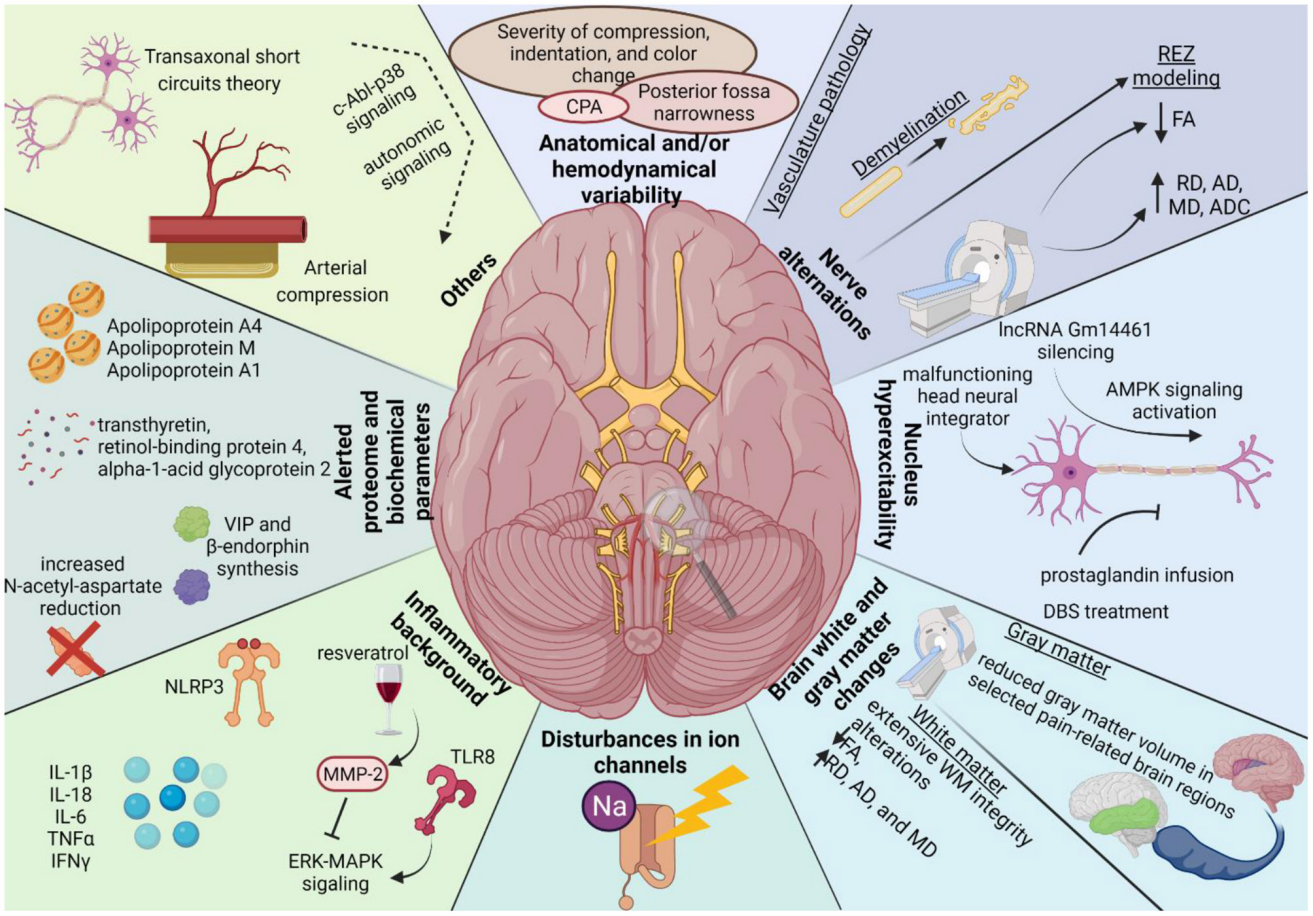
The graphical summary of the underlying pathogenesis of neurovascular compression syndromes based on performed systematic review. Legend as follow: AD, axial diffusivity; ADC, apparent diffusion coefficient; AMPK, adenosine monophosphate-activated protein kinase; CPA, cerebellopontine angle; FA, fractional anisotropy; MD, mean diffusivity; MMP-2, metalloproteinase-2; NLRP3, nucleotide-binding domain (NOD)-like receptor protein 3; lncRNA, Long non-coding RNA; RD, radial diffusivity; REZ, root entry/exit zone; WM, white matter.

Kim et al. evaluated the effects of selected parameters of the vertebral artery (severity of compression, indentation, and color change) on surgical treatment outcomes among HFS patients (see Figure 2). They revealed that patients with severe compression have slightly poorer outcomes than those with mild or moderate compression. Moreover, they concluded that CNVII color changes could be related to poor outcomes after MVD (Kim et al., 2012). Other important parameters affecting HFS patients are the angulation and tortuosity of vessels. Computational fluid dynamics models have confirmed that these lead to significant pressure differences between the vascular walls on opposite sides (Wang et al., 2019a).

Another hemodynamic parameter affecting HFS patients is higher pressure at the REZ. Zhang et al. found strong positive correlations between measured intraoperative pressure readings in NVC and (a) preoperative spasm severity according to Cohen scores and (b) the time to complete recovery after MVD (Zhang et al., 2019).

### Other Hemodynamical Factors Predisposing to NVC

Interestingly, TN can be caused by arterialization of the superior petrosal vein in the context of a dural or cerebral arteriovenous shunt (Robert et al., 2015). Robert et al. observed this arterialization in 10 (100%) patients with TN, while superior petrosal vein ectasia explained the compression of the CNV in two (20%). This could suggest that venous reflux rather than nerve compression is the primary mechanism of TN in this group of patients (Robert et al., 2015).

## Factors Associated With the Changes in the Nerve Structure

Selected factors associated with the affected nerve (e.g., length and volume of its central myelin portion) correlated strongly with the incidence of symptomatic NVC (Guclu et al., 2011). In this section, we collected NVC-related changes in the affected nerve: vasculature pathology, demyelination, REZ modeling, and alerted nerve structure revealed by MRI/diffusion tensor imaging (see Figure 2).

### Vasculature Pathology

Marinkovic et al. considered possible pathological vasculature among TN patients (*n* = 8; two with confirmed vascular compression). They examined biopsy specimens obtained during partial rhizotomy by electron microscopy and immunohistochemistry and found vascular pathological alterations in three of the patients, including the two with confirmed vascular compression. Electron microscopy revealed signs of apoptosis and/or degeneration of endothelial and smooth muscle cells in the trigeminal arteriole wall. Immunohistochemical tests gave stronger than normal reactions against factor VIII, laminin, fibronectin, and collagen IV, but weaker against CD31, CD34, and α-smooth muscle actin, among TN patients (Marinković et al., 2007).

### Demyelination

A study of a rat model of HFS by Kuroki and Møller showed that both CNVII demyelination and vascular compression are needed to induce facial hyperactivity. In other words, they confirmed that close contact between a peripheral branch of the facial nerve and an artery facilitates the development of abnormal muscle response, but only if the facial nerve had previously been slightly injured at the point of the arterial contact. They also revealed that blocking neural conduction in the facial nerve proximal to the artificial vascular compression point abolishes the abnormal muscle contraction. This observation could suggest that the anatomical location of cross-transmission is central to vascular compression (Kuroki and Møller, 1994).

The demyelination component of CNV can be estimated using diffusion tensor MRI radial diffusivity (RD). This approach was employed by Willsey et al. to predict the recurrence of TN following MVD. They noted that normalized RD correlates negatively with the duration of symptoms, possibly a useful predictor of pain-free remission (Willsey et al., 2021). However, this should be juxtaposed with histopathological examination. Such evidence was presented by Marinković et al., in the form of detailed ultrastructural and immunohistochemical examinations of trigeminal axons surrounded by the peripheral type of myelin. They confirmed not only central zone myelin changes (e.g., deformation, thickening, demyelination, and remyelination), but also peripheral myelin alterations visible by electron microscopy (e.g., atrophy or hypertrophy, increased neurofilaments, loss of the myelin and occasional sprouting) (Marinković et al., 2009). Similar findings were described by Devor et al., who examined trigeminal root specimens obtained during MVD (*n* = 12) ultrastructurally. They observed the following pathological changes: demyelination (*n* = 6), a range of less severe myelin abnormalities (dysmyelination: *n* = 11), and the presence of excess collagen (*n* = 8). They also noted axonopathy and axonal loss and residual myelin debris (Devor et al., 2002).

### REZ Modeling

Luo et al. investigated glial plasticity in the REZ of a rat TN model induced by compression injury. Their results revealed that mechanical compression injury leads to the activation of various cells (especially oligodendrocytes, astrocytes, Schwann cells, and macrophages) in the TZ (Luo et al., 2019). These alterations in glial cell plasticity could result in TN.

Similar observations were made by Lin et al., who also examined rat models: a TN group with CNV root compression, a sham operation group, a group with TN plus selective histone deacetylase inhibitor injection into the REZ, and a group with TN plus vehicle injection into the REZ. Their results also suggested that chronic nerve root compression could lead to TN via alerted glial plasticity and the level of histone acetylation in the REZ (Lin et al., 2019).

### Alerted Nerve Structure

NVC-linked structural alterations are not always apparent on conventional imaging (DeSouza et al., 2014). Therefore, we focus here on the description of NVC-induced lesions, which can easily be observed in magnetic resonance (MRI) and diffusion tensor (DTI) imaging.

### Magnetic Resonance Imaging Findings

High-resolution MRI can be used to explore nerve microstructure among patients with NVC. Using this approach, Wang et al. observed a lower CNV volume among TN patients than controls (Wang et al., 2019b). This was confirmed by Cheng et al. in a prospective case-control study of 60 consecutive patients diagnosed with TN compared with 30 sex- and age-matched healthy controls. They observed a significantly lower mean volume of the affected CNV than in either the non-affected side or the controls (Cheng et al., 2017).

### Diffusion Tensor Imaging Findings

DeSouza et al. used DTI to measure fractional anisotropy (FA) as well as identify abnormalities of mean (MD), radial (RD), and axial (AD) diffusivity from the REZ of CNV. They observed the following nerve alternations among TN patients: lower FA and higher RD, AD, and MD than in the control group. The MD and RD alterations could indicate that neuroinflammation and/or edema is an important element in TN pathophysiology (DeSouza et al., 2014).

Even more interesting observations were made by Leal et al., who employed DTI for a pre- and post-operative comparative study 4 years after MVD. The preoperative observations were similar to those of DeSouza et al.: significantly lower FA on the affected side than on the unaffected side or in controls. Affected nerves had smaller cross-sectional areas and volumes than unaffected nerves or the controls. Moreover, the apparent diffusion coefficient (ADC) on the affected side was significantly lower than that on the unaffected side or in the control group (Leal et al., 2019). These observations accord with the study by Pang et al. (2019). The postoperative findings showed that MVD did not change the differences in FA, but the ADC normalized in the affected nerves after MVD (Leal et al., 2019).

### Other Findings

Another parameter related to the affected nerve structure is an indentation defined as a furrowed hole on the facial nerve. Ko et al. estimated that 47% of HFS patients had an indented CNVII. Moreover, those patients required a longer recovery time after MVD than HFS patients without CNVII indentation (Ko et al., 2021).

## Nucleus Hyperexcitability

Several recent studies suggest that nucleus hyperexcitability is an important component in the pathogenesis of NVC-related symptoms. The relative involvements of the vascular component and hyperexcitability differed among patients, indicating that something other than a vascular component is involved in HSF pathology (Hirono et al., 2014). HFS was associated with ectopic excitation and ephaptic transmission of the facial nerve (Nielsen, 1984; Møller and Jannetta, 1986). By measuring facial motor evoked potentials during the MVD procedure, Wilkinson et al. showed that the suppressive effects of desflurane were less pronounced on the spasm side than on the non-spasm side, supporting a mechanism of central pathophysiology for HFS (Wilkinson et al., 2017). Ishikawa et al. measured F waves to investigate antidromically-activated neurons of the facial motor nucleus before and after the MVD procedure. They showed that in some patients, despite a successful MVD procedure, the increased F waves and abnormal muscle response persisted for several months and the muscle response normalized only after the F waves decreased (Ishikawa et al., 1996, 1997). A study by Teresaka et al. corroborated this observation. They indicated a correlation between preoperative anticonvulsant therapy and delayed cure after MVD, indicating an important role for hyperexcitation of the facial nucleus in the pathogenesis of HFS (Terasaka et al., 2016). This observation suggested that patients who did not respond to the MVD procedure should not be re-operated until the hyperexcitability of the facial motor nucleus has disappeared. The aforementioned neurophysiological observations are consistent with the known clinical observation (HFS patients after MVD present minor facial spasms for a few days after surgery with slow alleviation), which they explain perfectly (Illingworth et al., 1996).

A few pathomechanisms have been proposed to explain the role of disturbed neural activation in NVC-related symptoms. Cai et al. described a mouse model in which silencing of lncRNA Gm14461 led to activation of the AMPK and Akt/mTOR signaling pathways, enhancing autophagy and decreasing astrocyte activation (Cai et al., 2020). In another study, non-invasive CNX stimulation caused by prostaglandin infusion led to a rise in glutamate and decreased trigeminal nociceptive stimulation in allodynic rats (Oshinsky et al., 2014). Interestingly, in patients with obsessive-compulsive spectrum symptoms, where there are abnormalities in the stratio-thalamocortical circuits, focal dystonia and HFS were highly represented (Mula et al., 2012).

**Figure 3:**
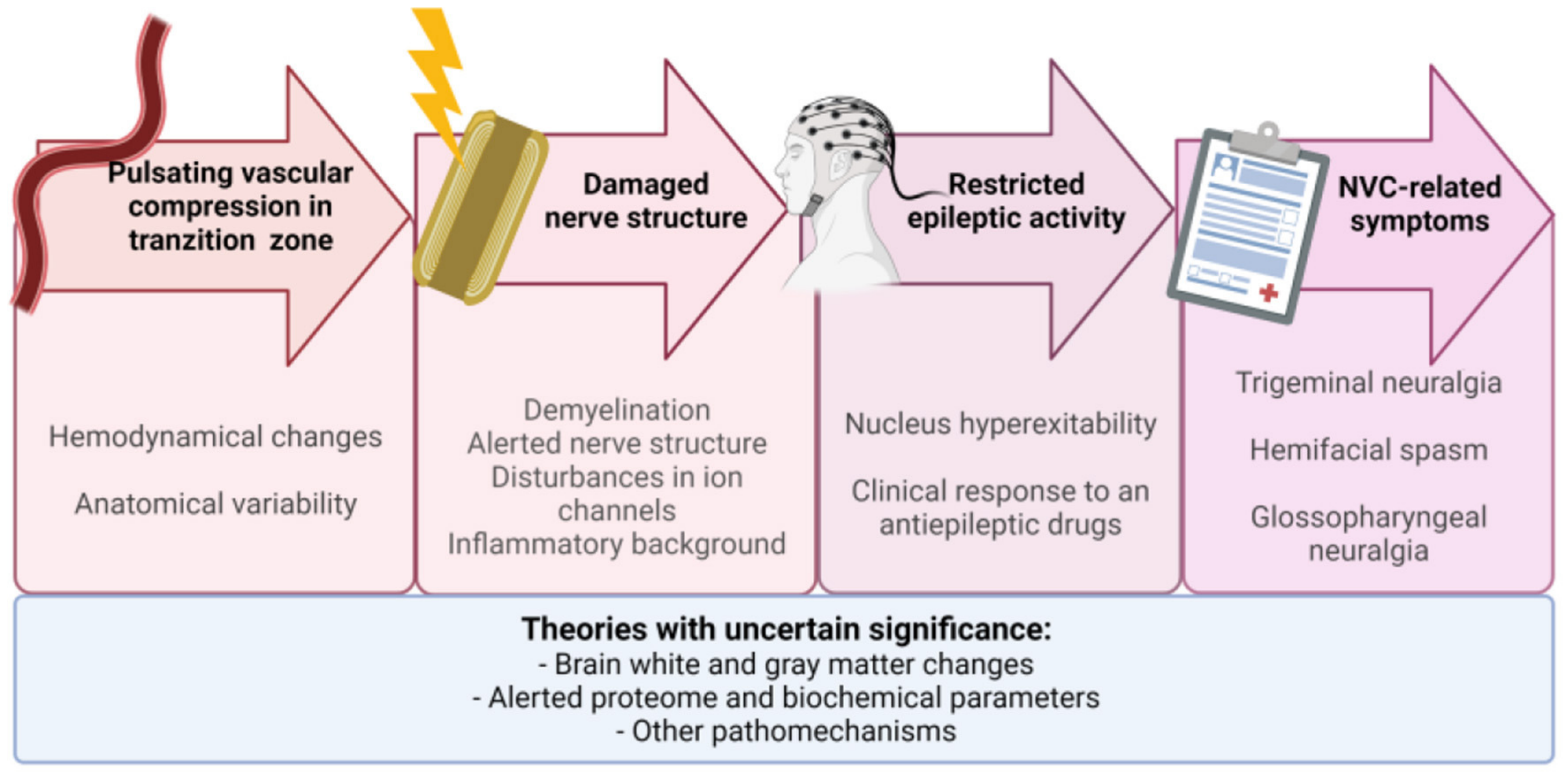
The proposed chain of events leading to neurovascular compression-related symptoms: vascular compression in transition zone → demyelination → nucleus hyperexcitability → symptoms. Not all factors mentioned in Figure 2 are presented here. Future research is needed to understand their role. NVC, neurovascular compression.

This phenomenon is somehow reminiscent of a restricted epileptic activity which might explain why, in the majority of cases, TN as well as the other NVC neuralgias well respond to an antiepileptic drug, namely to carbamazepine or gabapentin (see Figure 3). However, one must be aware that these medications are not helpful in HFS and other non-neuralgic NVCs.

## Brain White and Gray Matter Changes

Using high-resolution MRI, Wang et al. observed reduced gray matter volume in selected pain-related brain regions (e.g., insula, secondary somatosensory cortex, hippocampus, dorsal anterior cingulate cortex, precuneus, and several areas of the temporal lobe) (Wang et al., 2019b). Wu et al. compared morphological images of TN patients' brains with and without NVC to those of healthy controls. They employed voxel-based and surface-based morphometry to analyze whole-brain gray matter quantitatively. They observed significantly lower gray matter volume and cortical thickness in TN with NVC than in TN without NVC (Wu et al., 2020).

Moreover, they observed extensive alterations in white matter integrity (Wu et al., 2020). DeSouza et al., using diffusion tensor imaging data, also found abnormalities in brain white matter: lower FA, and higher RD, AD, and MD. These changes were especially visible in the corpus callosum, cingulum, posterior corona radiata, and superior longitudinal fasciculus (DeSouza et al., 2014).

## Disturbances in ION Channels

Many studies have highlighted the effect of disturbances in ion channels on neuropathic pain. This resembles the disturbances observed among NVC patients (Charlesworth et al., 2012; Wei et al., 2013; Choi et al., 2016; Li et al., 2021; Romero et al., 2021). Interestingly, the relationships between disturbances in ion channels and NVC have been explored in a few papers. Xia et al. observed overexpression of Nav1.8 in injured peripheral nerves. These molecular abnormalities were associated with the generation of HFS (Xia et al., 2014). Liu et al. used a rat infraorbital nerve chronic constriction injury model to test the character of TN as an ectopic impulse induced by a sodium channel modulated by cytokines. Nav1.3 was upregulated in the compressed CNV (Liu et al., 2020). Finally, functional testing of a mutation observed among NVC patients, TRPM8 (NM_024080.5):c.89G>A (p.Arg30Gln), revealed that it enhances channel activation and increases the basal current amplitude and intracellular calcium concentration, which could lead to TN (Gualdani et al., 2021). In the context of the hypothesis made in chapter 5, the disturbances in ion channels may be considered a functional variant of “damaged nerve” in the proposed chain of events leading to NVC symptoms.

## Inflammatory Background of Symptomatic NVC

Inflammatory cytokines induced by demyelination are known to cause neuropathic pain (Liu et al., 2019). Moreover, sticky inflammatory arachnoid membranes in the area of the NVC were a common finding during MVD (Chen et al., 2019a; Mazzucchi et al., 2019). Those observations suggested that an inflammatory response could be relevant to NVC-related syndromes.

An animal model revealed that the expression of a nucleotide-binding oligomerization domain containing a leucine-rich repeat and pyrin domain (NLRP), a type of NOD-like receptor belonging to innate immunity proteins, effectively modulates TN (Ren et al., 2021). Moreover, Chen et al. showed that injection of complete Freund's adjuvant to induce trigeminal pain leads to increased expression of the mRNAs for NLRP3, interleukin (IL)-1b, and IL-18. Further, NLRP3, IL-1b, and IL-18 expression was significantly inhibited by microRNA-186, which alleviated symptoms of neuralgia in CFA-treated mice (Chen et al., 2018). Accordingly, Liu et al. showed that upregulation of Nav 1.3 in TN seems to occur via an increase of IL-6 following constriction injury. Zhao et al. showed that expression of Toll-like receptor 8 (TLR8) is increased in trigeminal neurons in a mouse model of trigeminal neuropathic pain. In addition, deletion or knockdown of Tlr8 reduced the activation of ERK and p38-MAPK, decreased the expression of proinflammatory cytokines, and alleviated the neuralgia (Zhao et al., 2021). Liu et al. indicated that the purinoceptor P2X4, an essential receptor for preventing allodynia, could be involved in migraine chronicity via brain-derived neurotrophic factor-tyrosine receptor kinase B signaling following repeated inflammatory stimulation. Interestingly, resveratrol, a natural component of red wine, inhibited chronic constriction injury-induced allodynia via activation of matrix metalloproteases-9/2 and reduction of MAP kinase phosphorylation (Yang et al., 2016; Yin et al., 2019).

These observations were corroborated by studies on patients. Serum levels of IL-1β, IL-6, IL-8, TNF-α, white blood cell count, and neutrophil count in patients with HFS or TN were significantly higher than controls (Liu et al., 2018; Chen et al., 2019a). Ericson et al. used a proximity extension assay to analyze the levels of 92 inflammation-related protein biomarkers in cerebrospinal fluid from patients before and after microvascular decompression. They showed that surgery leads to a significant decrease of the immunological proteins to a level comparable with healthy controls (Ericson et al., 2019). Moreover, a study by Goebel et al. revealed differences in concentration of interferon-gamma between sample locations on the trigeminal nerve, highest in the distal zone (Goebel et al., 2018).

## Alerted Proteome and Biochemical Parameters

Recent studies have shown that the concentrations of several proteins in serum and cerebrospinal fluid of patients with NVC-related symptoms are distinctively different from healthy controls. Expressions of transthyretin, retinol-binding protein 4, and alpha-1-acid glycoprotein 2 among TN patients were higher than in the control group (Farajzadeh et al., 2018). Federico et al. showed by single-voxel proton magnetic resonance spectroscopy that patients with idiopathic spasmodic torticollis have a greater reduction of N-acetyl-aspartate in the basal ganglia, suggesting neuronal impairment in this region (Federico et al., 2001). The concentrations of high-density lipoproteins such as apolipoproteins A4, M, and A1 were increased in the cerebrospinal fluid of TN patients (Hamdeh et al., 2020). Moreover, Chen et al. showed that treatment with botulinum toxin type A inhibited the synthesis of vasoactive intestinal peptide and increased the synthesis of beta-endorphin, indicating a role for these proteins in the pathogenesis of TN (Chen et al., 2019b).

## Other Pathomechanisms

Besides the abovementioned studies related to the pathomechanism of NVC, a few studies have indicated that this pathology could have a different nature. According to the transaxonal short circuits hypothesis, squeezing nerve fibers together leads to the destruction of myelin sheaths and results in a transaxonal spread of impulses between neighboring neurons (Hankinson and Wilson, 1976; Kim and Fukushima, 1984; Kameyama et al., 2016). Accordingly, Truini et al. observed an association between demyelinating plaque, neurovascular compression, and TN in patients with multiple sclerosis (Truini et al., 2016). Nevertheless, it should be highlighted that MVD is considered as ineffective among MS patients presenting symptoms from NVC spectrum. Another mechanism was proposed in a study by Chen et al., who suggested that trigeminal nerve compression has an arterial rather than a venous origin (Chen et al., 2014). Another study indicated that c-Abl-p38alpha signaling mediates dopamine neuron loss and further TN (Fu et al., 2020). On the other hand, Zhou et al. proposed a role for disturbed signaling of the autonomic nervous system in the HFS. They showed that neurotransmitters released from autonomic nerves can induce action potentials in demyelinated nerves and could trigger HFS (Zhou et al., 2012). These insights should be reconsidered in the context of further research to avoid bias in their interpretation.

## Conclusions

Neurovascular compression syndromes remain challenging disorders for neurosurgeons, clinical neurologists, and scientists. Their underlying pathogenesis is not clear. There are many partial explanations. Specific anatomical and/or hemodynamical changes alone seem insufficient to account for NVC-related symptoms. Such anatomical variability must coexist with additional changes such as factors associated with affected nerve (e.g., demyelination, REZ modeling), nucleus hyperexcitability, brain white and/or gray matter changes, or disturbances in ion channels. Moreover, the effects of inflammatory background alerted proteome, and biochemical parameters on symptomatic NVC cannot be ignored. Further studies are needed to gain better insight into NVC pathophysiology.

## Practice Points

- Clinically, we can distinguish the following neurovascular compression (NVC) conditions: trigeminal neuralgia, hemifacial spasm, and glossopharyngeal neuralgia. Also, rare cases of geniculate neuralgia and superior laryngeal neuralgia are reported. Other syndromes, e.g., disabling positional vertigo, hypertension in the course of NVC at the CN IX-X root entry/exit zone, and torticollis, have insufficient clinical evidence for microvascular decompression.
- NVC syndromes remain challenging disorders. The exact pathomechanism leading to characteristic NVC-related symptoms remains unclear. The most likely chain of events leading to NVC symptoms is vascular compression in transition zone → demyelination → nucleus hyperexcitability → symptoms.
- The root entry/exit zone should not be equated with the transition zone (TZ) between the central and peripheral myelin. This observation has a significant clinical consequence. During the surgery, it is important to visualize the compression of an appropriate vessel on the nerve in the TZ. When the TZ is retracted toward the pons, the neurosurgeon should look for conflict between vessel and pons rather than nerve.

## Data Availability Statement

The original contributions presented in the study are included in the article/Supplementary Material, further inquiries can be directed to the corresponding author/s.

## Author Contributions

Conceptualization: BS, MR, and DJJ. Literature search and manuscript preparation: BS and JS. Revision of the manuscript: MB, JJ, PPL, DJJ, GW, FKK, AG, MS, RST, and MR. All authors read and approved the final manuscript.

## Conflict of Interest

The authors declare that the research was conducted in the absence of any commercial or financial relationships that could be construed as a potential conflict of interest.

## Publisher's Note

All claims expressed in this article are solely those of the authors and do not necessarily represent those of their affiliated organizations, or those of the publisher, the editors and the reviewers. Any product that may be evaluated in this article, or claim that may be made by its manufacturer, is not guaranteed or endorsed by the publisher.

## Abbreviations

AD: axial diffusivity
ADC: apparent diffusion coefficient
CPA: cerebellopontine angle
DBS: deep brain stimulation
FA: fractional anisotropy
HFS: hemifacial spasm
IL: interleukin
MD: mean diffusivity
MVD: microvascular decompression
NLRP, Nucleotide-binding oligomerization domain: Leucine-rich Repeat and Pyrin domain-containing
NVC: Neurovascular compression syndromes
RD: radial diffusivity
REZ: root entry/exit zone (also known as Obersteiner-Redlich zone)
TLR8: Toll-like receptor8
TN: trigeminal neuralgia
TZ: transition zone
WM: white matter.
