# Supplementary material for: The Underlying Pathogenesis of Neurovascular Compression Syndromes: A Systematic Review
Source: Front Mol Neurosci. 2022 Jul 4;15:923089. doi: 10.3389/fnmol.2022.923089 (PMC9289473; doi:10.3389/fnmol.2022.923089)
Supplement: Supplementary file 1 [file Presentation_1.pdf]

Supplementary Material

1 Search Details:

1.1 PubMed

("neuro-vascular conflict" OR "neuro-vascular conflicts" OR "neurovascular conflict" OR "neurovascular conflicts" OR "neurovascular compression syndrome" OR "neurovascular compression syndromes" OR "nerve cascular compression syndrome" OR "nerve cascular compression syndromes" OR "trigeminal neuralgia" OR "hemifacial spasm" OR "disabling positional vertigo" OR "torticollis") AND ("pathogenesis" OR "pathogenesis" OR "etiopathogenesis" OR "pathomechanism" OR "mechanism" OR "pathophysiology")

1.2 Web of Science:

("neuro-vascular conflict" OR "neuro-vascular conflicts" OR "neurovascular conflict" OR "neurovascular conflicts" OR "neurovascular compression syndrome" OR "neurovascular compression syndromes" OR "nerve cascular compression syndrome" OR "nerve cascular compression syndromes" OR "trigeminal neuralgia" OR "hemifacial spasm" OR "disabling positional vertigo" OR "torticollis") AND ("pathogenesis" OR "pathogenesis" OR "etiopathogenesis" OR "pathomechanism" OR "mechanism" OR "pathophysiology")
